# OphthoACR (Ophthalmology Automated Chart Review): An AI-Powered Tool for Complete Automation of Ophthalmology Chart Reviews and Cohort Data Analysis

**DOI:** 10.1167/tvst.14.10.8

**Published:** 2025-10-09

**Authors:** Karen M. Chen, Kevin W. Chen, Vlad Diaconita, Stanley Chang, Leejee H. Suh

**Affiliations:** 1 Columbia University Irving Medical Center, Department of Ophthalmology, New York, New York, USA; 2 Department of Ophthalmology, Louisiana State University Health Science Center, New Orleans, Louisiana, USA

**Keywords:** artificial intellgence, chart review, automated chart review, large cohort analysis, secondary IOL

## Abstract

**Purpose:**

Retrospective chart reviews in ophthalmology are essential for gaining clinical insights, but they remain labor-intensive and prone to error. Despite digitization through electronic health records, extracting and interpreting lengthy, unstructured patient histories remains challenging, particularly in ophthalmology, which relies heavily on both imaging and text-based reports. We introduce OphthoACR, a Health Insurance Portability and Accountability Act–compliant artificial intelligence (AI)–powered tool for automated chart review and cohort analyses in ophthalmology.

**Methods:**

OphthoACR was applied to extract 16 variables of increasing task difficulty from the complete chart histories of 91 patients who underwent secondary intraocular lens surgery at the Columbia University Irving Medical Center from January 2020 to August 2024, for a total of 5834 unique documents. The tool integrates a fine-tuned large language model into a robust pipeline to extract and contextualize unstructured clinical data, including operative reports and imaging documents. OphthoACR's performance was compared to manual and AI-assisted chart reviews.

**Results:**

OphthoACR achieved 94% accuracy in extracting variables of interest, significantly outperforming manual review (83%). It demonstrated 97% specificity, 92% sensitivity, and a Cohen's κ of 0.70, indicating robust agreement. Average time for OphthoACR to process a patient chart was 80 seconds, a 95% reduction compared to the manual review's average of 25.2 minutes. For cohort-wide processing, the improvement was 99.9% due to parallel processing of patients’ charts.

**Conclusions:**

OphthoACR significantly improves the accuracy and efficiency of ophthalmology chart reviews, offering an unprecedented automated solution to analyze large patient cohorts.

**Translational Relevance:**

OphthoACR provides end-to-end automation of retrospective chart reviews, transforming the currently labor-intensive manual process into an efficient, accurate, and scalable solution that substantially enhances clinical research.

## Introduction

Artificial intelligence (AI) has gained significant traction in health care in recent years, offering transformative potential to streamline clinical workflows and enhance patient outcomes. One area that particularly benefits from AI's capabilities is retrospective chart review. Despite the widespread digitization of patient records through electronic health records (EHRs) in the past decade, efficient extraction and interpretation of large volumes of unstructured clinical notes remain a significant challenge.^1^ The field of ophthalmology faces additional complexities due to its heavy reliance on the multimodal nature of documentation sources, including surgical reports, imaging results, and handwritten records. Traditional chart reviews in ophthalmology are not only heavily time-consuming but also vulnerable to human error.^2^ Automated analysis of chart data is rapidly becoming a key priority for health care institutions, as demonstrated by Johns Hopkins Hospital's $5 million annual budget allocated for EHR-related tasks.^3^

In the past decade, AI tools, including named entity recognition and natural language processing (NLP) models, have shown promise in extracting structured data, such as visual acuity (VA) and intraocular pressure (IOP), but have continued to struggle with capturing nuanced and context-dependent information like postoperative complications embedded within free-text notes.^4^^–^^7^ With the rise of large language models (LLMs), such as OpenAI's GPT series and Meta's LLaMA, the unprecedented capabilities of these models are gaining traction due to their wealth of pretrained knowledge and advanced ability to analyze human text. Despite these advances, a fully automated solution for end-to-end chart review in ophthalmology remains a challenge.

This study introduces OphthoACR (Ophthalmology Automated Chart Review), a multimodal Health Insurance Portability and Accountability Act (HIPAA)–compliant AI-powered agent that represents the first fully automated solution for end-to-end chart review in ophthalmology. OphthoACR is not just an analysis tool but acts as a full agent, featuring a fine-tuned LLM integrated into a custom pipeline that manages and automates all aspects of patient chart reviews and cohort analyses. It efficiently extracts and interprets key variables like surgical details, ocular parameters, and complications from EHR charts, handwritten notes, and imaging reports. By automating each step of the retrospective chart review process, including preprocessing, data extraction, output cleaning, and cohort analysis, OphthoACR dramatically reduces processing time from weeks or months in manual review to minutes, setting a new standard for comprehensive ophthalmology chart reviews.

## Methods

This section describes the development of OphthoACR, including the preprocessing, model training, and postprocessing stages. In addition, we outline the methodology used to evaluate the tool's effectiveness by comparing the model's outputs to the results of traditional manual and AI-assisted chart reviews.

### HIPAA Compliance and Data Security Infrastructure

In the United States, HIPAA sets national standards for protecting sensitive patient health information, ensuring its confidentiality, integrity, and availability. OphthoACR was developed and deployed in full compliance with HIPAA regulations.

All components of the OphthoACR pipeline—including data storage, LLM processing, and execution environments—operate within a HIPAA-compliant infrastructure. HIPAA-compliant use of OpenAI's GPT-4 LLM is specified in the OpenAI–Columbia University Business Associate Agreement (BAA), a legal contract required under HIPAA for entities handling protected health information (PHI) on behalf of a covered entity. The BAA outlines responsibilities to safeguard PHI and ensures compliance with HIPAA's privacy and security rules.

Access to Columbia University's HIPAA-compliant OpenAI API is controlled via a unique API key issued by the university’s IT department, ensuring only approved users can interact with the API. To further safeguard sensitive information, users are required to establish encrypted access to the EHR system through Columbia University's secure network. Data retrieval is strictly controlled: users submit a list of medical record numbers (MRNs) in compliance with an institutional review board–approved study protocol. Extracted data are securely stored on encrypted, HIPAA-compliant servers in alignment with institutional data safety policies. All pre- and postprocessing scripts are executed on HIPAA-compliant endpoints, ensuring end-to-end adherence to federal standards for privacy, security, and PHI management.

### OpenAI Privacy and Security Infrastructure

 OphthoACR's privacy and security are further supported by OpenAI's robust infrastructure. OpenAI's API platform has been audited and certified for security operations center (SOC) two compliance and evaluated by an independent third-party auditor to confirm alignment with industry standards for security and confidentiality. OpenAI encrypts data at rest using AES-256 and in transit using TLS 1.2+, ensuring protection during all stages of data handling. Importantly, OpenAI does not use data submitted via ChatGPT Education to train or improve its models, and no third parties have access to this information.

Comprehensive documentation on OpenAI's privacy, security, and HIPAA compliance is publicly available at https://trust.openai.com. Details on Columbia University and OpenAI's BAA agreement may be found at https://www.cuit.columbia.edu/content/chatgpt-education.

### Implementation Requirements for OphthoACR

OphthoACR must be run within a HIPAA-compliant computing environment to ensure secure handling of protected health information. Running the tool requires access to a HIPAA-compliant OpenAI API environment, which is enabled through a BAA between the institution and OpenAI. Once the BAA is in place, the institution provides researchers with a HIPAA-compliant API key that grants secure access to the GPT-4 API. The pipeline can then be run locally on a standard laptop or workstation with Python (≥3.8) installed. No GPU or high-performance computing infrastructure is required. Users can execute the pipeline using any standard code editor, such as Jupyter Notebook or Visual Studio Code. Internet access is required to communicate with the OpenAI API using the provided API key. OphthoACR was created using OpenAI's GPT-4 model gpt-4o-2024-08-06 and text-embedding-ada-002 for retrieval-augmented generation. Python libraries used in OphthoACR include openai, pandas, numpy, tqdm, regex, and PyPDF2, with optional support for optical character recognition (OCR) tools such as OpenCV or Tesseract if scanned documents are used. These minimal hardware and software requirements make OphthoACR easily deployable in a wide range of clinical and research environments.

### Study Design and PreProcessing of Patient Chart Histories

A total of 91 patients at Columbia University Irving Medical Center (CUIMC) Harkness Eye Institute, who underwent secondary intraocular lens (IOL) surgery from January 2020 to August 2024, were identified using Current Procedural Terminology code 66985. All ophthalmology records, including EHR notes, scanned handwritten notes and charts, and imaging results, were packaged for the input and development of OphthoACR, for a total of over 5834 multimodal documents. Identifying information, including MRNs and full names, was systematically removed from the records using exact matching, regex-based patterns, and fuzzy matching techniques, consistent with the safe harbor method. Secondary IOL surgery was specifically chosen to evaluate OphthoACR's robustness on ophthalmic data due to the procedure's inherent complexity and the typically extensive preexisting ocular histories of these patients, which often include multiple comorbid eye diseases, thereby ensuring that the cohort encompassed a wide breadth of ophthalmic conditions. The OphthoACR pipeline was run using “gpt-4o-2024-08-06.”

### Testing of OphthoACR on Tasks of Varying Complexity

OphthoACR was evaluated on 16 tasks categorized by complexity, ranging from simple data extraction to advanced interpretation of unstructured text (see Supplementary Materials). Simple tasks included extracting straightforward surgical parameters, such as the operated eye and lens type, while intermediate tasks required tracking visual parameters like VA, axial length, and IOP across multiple time points, including at 3 months postoperatively. Advanced tasks involved identifying postoperative complications, defined as abnormalities that persisted beyond 3 months after surgery and were often described inconsistently or in unstructured formats (Supplementary Fig. S1). These tasks were difficult and previously unable to be accomplished by chart review tools based on NLP or feature matching methods due to the need for logic and interpretation.^8^ For intermediate and advanced tasks, text was segmented and processed iteratively to address context length limitations. Additional parameters can be seamlessly integrated into OphthoACR by defining new variables of interest.

### Ground-Truth Labels

Ground-truth labels, defined as the accurate “gold-standard” value to which our AI-extracted output was compared, were established through consensus among three clinical reviewers experienced in ophthalmic reports. Ophthalmic surgical details and ocular values at 3 months postoperation were manually extracted, and complications were noted as either present or absent.

### Preprocessing of Digitized Charts and Scanned Paper Documents for OphthoACR

Each patient's ophthalmology chart history—including structured data from the electronic health record (Epic), imaging results, and scanned legacy documents such as handwritten notes and ocular measurement reports—was compiled into a digital folder labeled by patient ID number. Scanned paper records and image-based content were preprocessed using OpenCV to enhance contrast, brightness, and clarity in order to facilitate accurate OCR. OCR was performed using GPT-4 Vision to convert all image data into machine-readable free text.

The resulting free-text data, including both EHR exports and OCR-converted text content, were then subjected to systematic preprocessing via a custom Python script (see Supplementary Materials). This script standardized the format and language of the text across all patient records to increase overall consistency between charts. Key preprocessing steps included the following:

1. Removal of extraneous white space and line breaks: “OD: 20/40 \n\n\n \n \n \n\n OS: 20/60” → “OD: 20/40 OS: 20/60”
2. Normalization of medical terminology and abbreviations: “IOP: R 15, L 16” or “Tn: 15/16” → standardized to “Intraocular Pressure: OD 15 mmHg, OS 16 mmHg” “CME” → “Cystoid Macular Edema”
3. Standardization of tabular data (e.g., intraocular pressure charts, visual acuity logs) to improve machine readability

This process ensured that both structured and unstructured data across various documentation sources were rendered into a coherent, more standardized formats suitable for successive large-scale LLM analysis.

### Model Development

OpenAI's GPT-4 API served as the base LLM for OphthoACR, and its parameters, including temperature, were optimized for coherence, diversity, and processing capacity. The implemented parameters were temperature = 0.1, *n* = 5, top_p = 0.9, frequency_penalty = 0.2, presence_penalty = 0, and model = “gpt-4o-2024-08-06.” To enhance the model's ability to extract clinically relevant ophthalmic data, we employed retrieval-augmented generation (RAG) and fine-tuning.

### Retrieval-Augmented Generation

To tailor GPT-4 for clinical ophthalmology data extraction and interpretation, we implemented a RAG pipeline designed to contextualize model responses with patient-specific data. The pipeline consisted of three primary stages: segmentation, indexing, and retrieval.

### Segmentation and Preprocessing

Each patient's compiled chart (as described above) was parsed into discrete data blocks, segmented by document type and content domain. Structured data fields—such as IOP, VA, medication lists, IOL power, laterality, and surgical dates—were extracted using a combination of rule-based regular expressions and custom-built domain-specific parsers tailored for ophthalmologic documentation. Input sources included both structured EHR fields and unstructured clinical notes that contained embedded structured elements (e.g., vitals sections, preoperative checklists, imaging summaries). Segmentation was performed by identifying section headers (e.g., “Assessment and Plan,” “Operative Report,” “Postoperative Course”) using predefined header variants. Each document was divided into semantically discrete blocks using a sliding window tokenizer (512-token chunks with 100-token overlap) to preserve context. Structured elements within each block were parsed using field-specific templates (e.g., IOP patterns such as “IOP: OD 14, OS 16” or VA entries like “VA (sc): 20/80 OD, 20/100 OS”) and normalized into a standardized schema. Ambiguous cases (e.g., unlabeled laterality or inconsistent unit usage) were resolved using contextual cues, including from surrounding text, and categorized into a respective header type. Each chunk was tagged with metadata, including patient ID, date, document type, and section header.

### Indexing

All segmented data blocks were embedded into high-dimensional vector representations using OpenAI's text-embedding ada-002 model. These embeddings were stored in a Facebook AI Similarity Search (FAISS) index to support fast, similarity-based retrieval. Embeddings were linked to their original text chunks and metadata to allow contextual reassembly during downstream processing.

### Retrieval and RAG Integration Into Prompt Construction

During inference, user queries (e.g., “summarize the patient's response to aflibercept injections”) were embedded and used to retrieve the relevant chart segments from the FAISS index based on cosine similarity. Retrieved segments were dynamically incorporated into the model's input prompt. This prompt assembly ensured that GPT-4 responses were grounded in real patient data rather than general domain knowledge. To maximize fidelity and reduce hallucination risk, we used sampling (*n* = 5), which we later used to filter based on output confidence and factual overlap with source data. This pipeline allowed OphthoSearch to generate context-aware, clinically accurate outputs across heterogeneous ophthalmology records while maintaining traceability to source documentation.

### Fine-Tuning

To improve GPT-4’s capacity for extracting ophthalmology-specific clinical data, the model was fine-tuned using a custom data set of 10,000 input–output pairs derived from institutional surgical notes and biomedical literature. A total of 4000 examples were curated from operative and postoperative notes from patient charts, covering procedures such as secondary IOL surgery, vitrectomy, and glaucoma shunt surgery documented between 2018 and 2023. Notes were selected based on the completeness of preoperative, intraoperative, and follow-up documentation. These records were annotated to extract clinically relevant endpoints with the assistance of keyword-matching techniques, including postoperative IOP, VA, lens type and power, and complications, such as macular edema and anterior chamber inflammation. Annotations were formatted as question–answer pairs or summarization tasks. To complement this clinical data set, 6000 biomedical literature samples were retrieved from PubMed via the NCBI Entrez API using targeted search queries related to ophthalmology and specific subtopics of interest, such as “postoperative complications AND secondary IOL surgery” and “visual outcomes AND intraocular lens.” These searches can be tailored to specific use cases. Included studies consisted of clinical trials, meta-analyses, and cohort studies reporting quantitative outcomes and complication rates. Input–output examples from both chart notes and literature were generated using a combination of automated keyword-based matching and manual validation to ensure clinical relevance, consistency, and accuracy. Relevant text was converted into structured prompts reflecting ophthalmic measurements, outcomes, and complication summaries. Fine-tuning was performed using supervised learning on the combined data set, with GPT-4 trained over four epochs using a batch size of 16, learning rate of 2e-5, and a weight decay parameter of 0.01 to reduce overfitting. Then we employed iterative optimization of model weights via gradient descent to minimize prediction error. Overall, fine-tuning enabled the model to better interpret ophthalmology-specific language and structured/unstructured data formats, thereby improving precision and reliability for automated chart review.

### OphthoACR Model Call and Output Generation

For each variable of interest, we developed a custom prompt to return variables in JavaScript Object Notation (JSON), a text-based format that is both human-readable and machine-parsable. Using OpenAI's Structured Outputs feature, we ensured that our model generates responses that adhere to our supplied JSON schema. In addition, we also employed few-shot prompting to provide additional context for the output. In few-shot prompting, a limited number of example inputs and outputs are provided to help guide the LLM and reinforce in-context learning.^9^ Lastly, as described in the RAG implementation, the retrieved content from the RAG pipeline is embedded into the prompt, enabling contextual grounding. An example prompt schema used with the LLM can be found in the Supplementary Materials.

For each patient, OphthoACR's fine-tuned model was called on each variable of interest. For intermediate and advanced variables, the results for each model call were appended to the JSON output, adding newly extracted data to the output with each iteration. This method of iterative extension ensures that the output becomes increasingly comprehensive, capturing all relevant clinical information from the patient's records. For each call to the model, multiple candidate outputs were generated by the model, and only the highest confidence output, as evaluated by the confidence heuristics detailed in the Supplementary Materials, was returned, using best-of-*n* (*n* = 5) sampling. This improves the overall quality of the output by allowing the model to generate multiple possibilities and select the most coherent, relevant, or accurate response, thereby reducing randomness and increasing the likelihood of getting a more optimal result.

### Post-Processing

OphthoACR was designed to generate structured, high-quality JSON outputs to ensure standardization, accuracy, and seamless integration into downstream clinical workflows. To achieve this, we implemented an iterative postprocessing pipeline that

1. Cleaned and validated JSON outputs to ensure accurate downstream analysis
2. Standardized extracted values to align with clinical benchmarks
3. Parsed out temporal individual outcomes and postoperative complications
4. Following the completion of the individuals’ chart reviews, conducted a cohort-wide analysis to provide immediate clinical insights

Detailed steps of the postprocessing pipeline may be found in the Supplementary Materials.

### Conducting Manual and AI-Assisted Manual Review

For the manual review process, all clinical variables as outlined above were independently extracted by an independent clinician.^5^ This involved a thorough examination of all data, including EHR notes and imaging reports from patient charts, by hand. Results were logged in Excel 2024 (Microsoft, Redmond, WA, USA). The AI-assisted manual review process integrated traditional manual chart review with outputs generated by OphthoACR to enhance both accuracy and efficiency in data extraction. OphthoACR provided structured outputs for each clinical variable, such as intraocular pressure trends, visual acuity trajectories, surgical dates, postoperative complications, and medication usage, along with the corresponding source text excerpts. These outputs were reviewed by a clinician, who used them to prioritize sections of the chart for verification and to cross-reference timelines of key clinical events. The clinician flagged any discrepancies between the AI-extracted values and their chart review.

### Statistical Comparison of AI Only Versus Manual Versus AI-Assisted Review

The performance of OphthoACR was evaluated against manual and AI-assisted manual review workflows across basic, intermediate, and advanced data extraction tasks. Performance metrics for binary classification tasks (e.g., identification of complications, medication use, or presence of imaging findings) included accuracy, sensitivity, specificity, and Cohen's κ to quantify interrater agreement between automated and reference-standard outputs. Time-based efficiency analyses compared median review times across manual, AI-assisted, and AI-only workflows using the Wilcoxon signed-rank test as well. All statistical analyses were conducted using Python libraries: Pandas (v1.5+), NumPy (v1.23+), Scikit-learn (v1.2+), and statsmodels (v0.13+).

## Results

**Figure 1.**
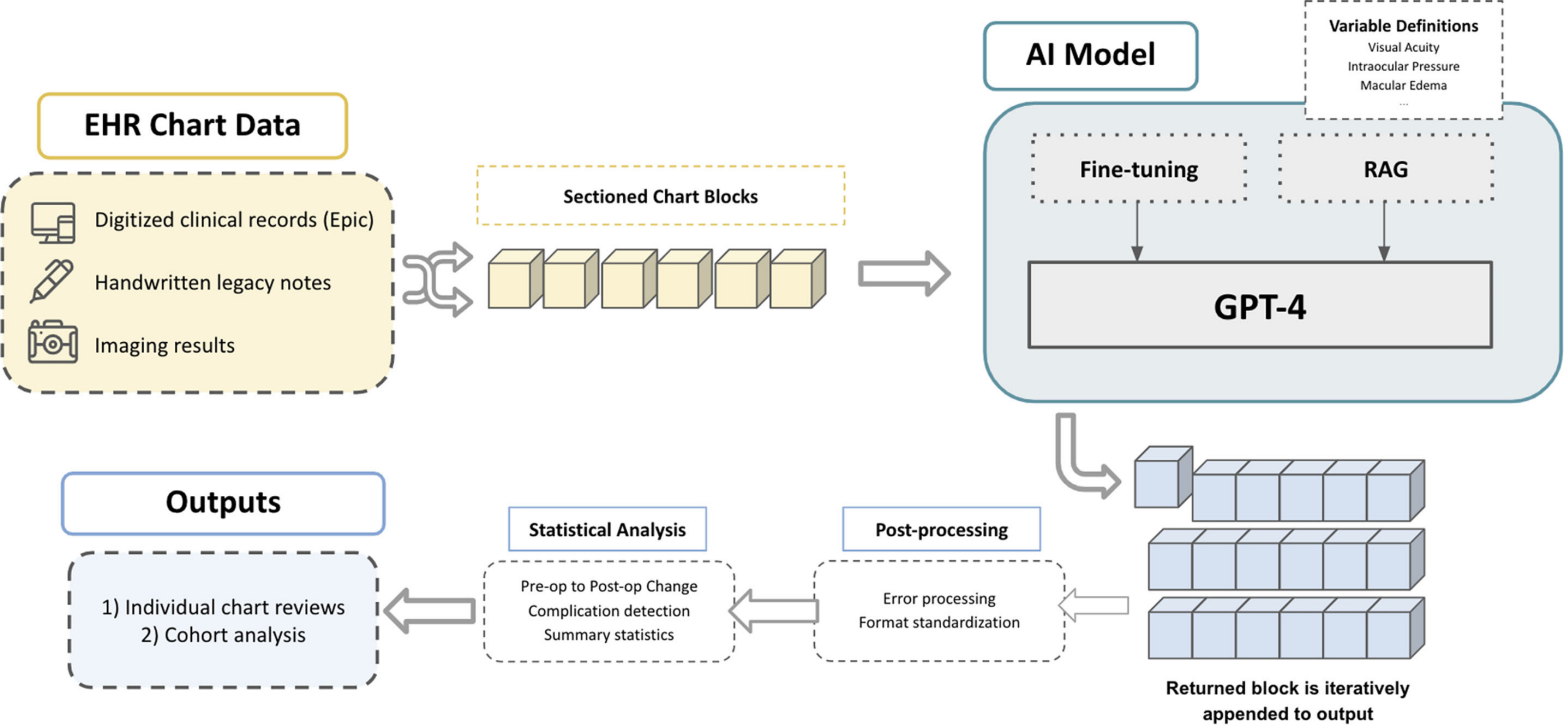
Pipeline development and execution of OphthoACR. EHR chart data are processed through OphthoACR's fine-tuned, RAG-enhanced LLM model, which generates refined final outputs.

**Figure 2.**
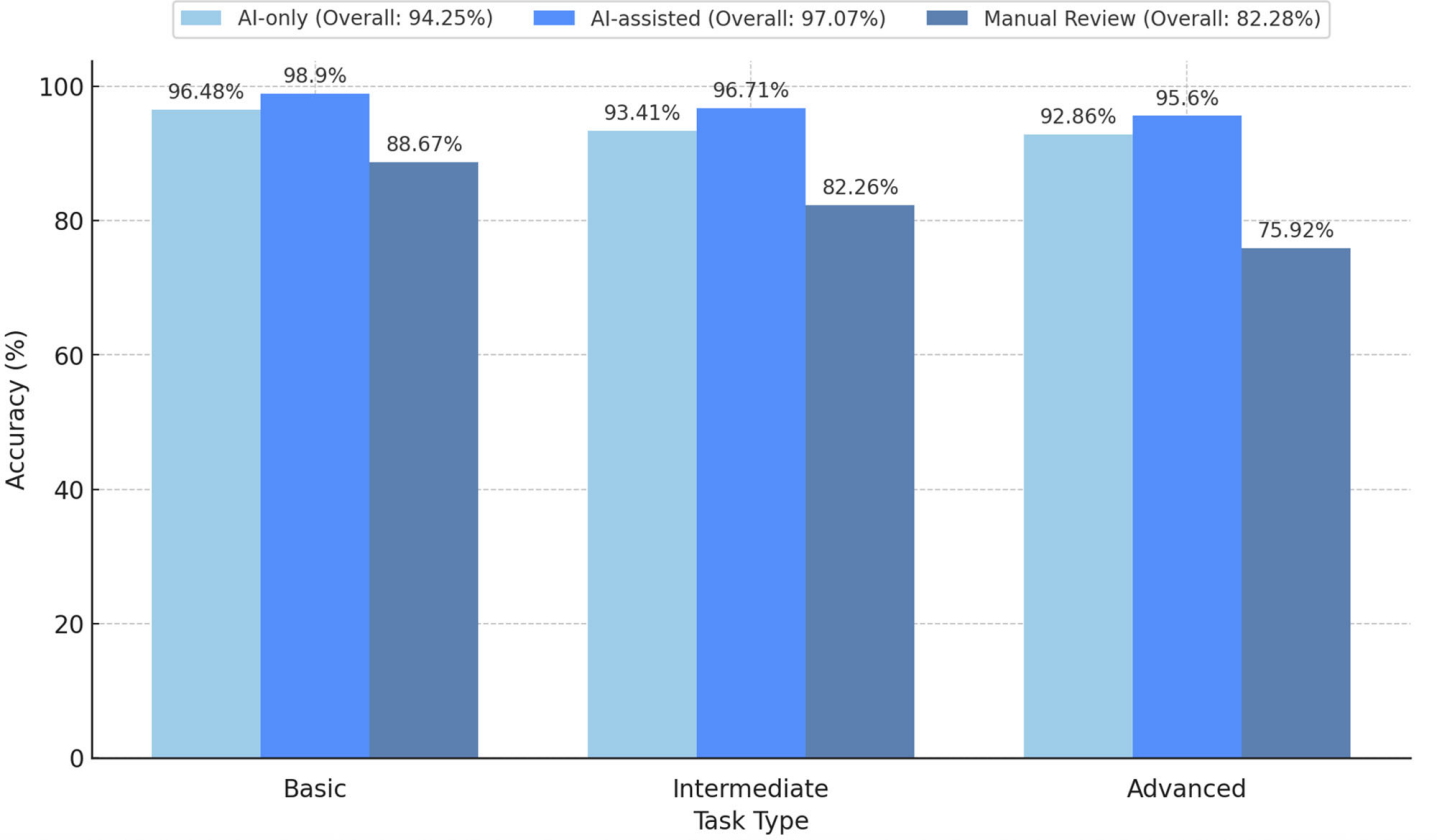
Accuracy of AI-only, AI-assisted, and manual chart review, categorized by task difficulty. Overall accuracy for each review type is notated in the legend.

**Figure 3.**
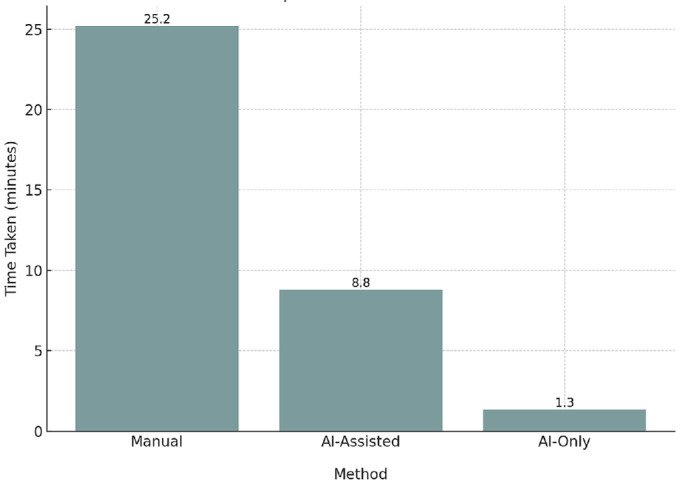
Average time (minutes) per chart taken for manual, AI-assisted, and AI-alone chart review for 21 variables of interest.

OphthoACR achieved an overall accuracy of 94.25%, with 96.48% accuracy for simple tasks (e.g., identifying lens models) and 92.62% for complex tasks like detecting postoperative complications (Fig. 1). In comparison to manual review, AI review improved accuracy by 12% while reducing per-patient review time by 94.8% and cohort-wide review time by 99.9% (Figs. 2, 3). These time savings do not account for the time spent on data preprocessing and deidentification prior to tool deployment.

The exceptional speed of cohort-wide review time is a result of the ability of OphthoACR to process each patient's data in parallel, allowing simultaneous analysis of an entire patient cohort. Therefore, the total processing time for OphthoACR is determined by the longest processing time of the single patient chart in the cohort, rather than the cumulative time it takes to analyze each patient in manual review, where each chart is processed sequentially. (This results in a total runtime of O(1) in AI review as opposed to O(n) in manual review.)

### OphthoACR Outputs: Individual-Level Chart Review and Cohort-Wide Summary Statistics

**Figure 4.**
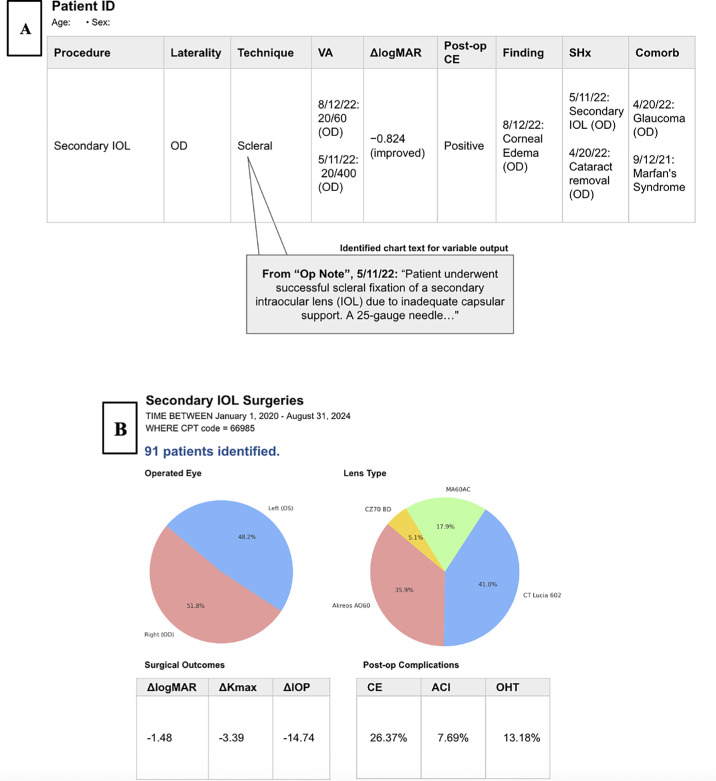
Abbreviated preview of an example individual-level chart review and cohort-wide analysis by OphthoACR. The JSON output format has been cleaned for clarity of viewing. (**A**) In individual-level chart review, variables of interest are returned with a snippet from the patient's chart that serves the model's justification for the output. All instances of temporal values are output. (**B**) Cohort-wide summary statistics, procedural outcomes, and complications are displayed for an identified cohort of patients.

For each patient, OphthoACR generates a structured data frame of JSON data, providing postprocessed, clinically usable variables without the need for extensive restructuring or manual cleaning (Fig. 4). Key outputs include pre- to postoperative changes in clinical parameters such as VA and IOP, detailed complication records with dates, and systematic documentation of other relevant diseases and surgeries, offering a comprehensive overview of the patient's clinical history. All extracted temporal variables, including ocular parameters and complications, are supplied with dates and have the option to be seamlessly plotted to view changes over time (Supplementary Fig. S2). Additionally, for each output, the model provides the location and snippet from the chart as justification, providing convenient oversight of the model's outputs. An abbreviated preview of the individual-level and cohort-wide summary statistics for select variables is presented in Figure 4.

### Performance of OphthoACR Automated Chart Review

Performance metrics for OphthoACR's data extraction compared to manual chart review are presented in Table 1. The pipeline achieved an overall accuracy of 94.25%, a sensitivity of 92.14%, and a specificity of 97.02%. It processed each chart in an average of 80 seconds, with the longest processing time reaching 243 seconds. For basic tasks, OphthoACR achieved 96.48% accuracy, ranging from 94.51% for lens model identification to 98.90% for determining the operated eye. Accuracy remained high for intermediate tasks (93.41%). Retrieving ocular parameters at the 3-month mark was more accurate (93.41%) when we postprocessed the comprehensive list of ocular parameter outputs generated through multiple runs of OphthoACR compared to directly prompting the LLM model for the value (61.16%).

**Table 1.** Performance Statistics of OphthoACR's AI-Automated Chart Review of 91 Patients Who Underwent Secondary IOL Surgeries Compared to Ground-Truth Values, Organized by Task Difficulty

	Model Performance by Task Difficulty					
Characteristic	IOL Model	Power	Technique	Date	Laterality	Average
Basic surgical characteristics						
Accuracy, %	94.51	95.60	95.60	97.80	98.90	96.48
Sensitivity, %	97.67	97.70	97.70	98.88	98.89	98.17
Specificity, %	97.15	98.56	98.56	98.56	100.00	98.56
	VA	AL	Kmax	IOP	Average	

Intermediate ocular parameters (3 months postoperatively)						
Accuracy, %	94.51	93.41	93.41	92.31	93.41	
Sensitivity, %	97.67	96.47	96.47	96.43	96.76	
Specificity, %	97.15	97.15	97.15	97.15	97.15	
	CE	ME	ACI	OHT	Average	

Advanced surgical complications (3 months postoperatively)						
Accuracy, %	89.01	93.82	95.06	92.59	92.62	
Sensitivity, %	86.41	84.90	72.78	81.87	81.49	
Specificity, %	92.53	95.83	97.34	95.65	95.34	
Cohen's κ	0.89	0.87	0.79	0.87	0.86	

ACI, anterior chamber inflammation; AL, axial length; CE, corneal edema; ME, macular edema; OHT, ocular hypertension.

For advanced tasks, including detecting postsurgical complications such as corneal edema, macular edema, anterior chamber inflammation, and ocular hypertension at 3 months postoperatively, OphthoACR maintained a strong overall accuracy of 92.62%. Of note, our recursively structured JSON extraction framework—incorporating schema enforcement, field-level validation, and consistency checks—achieved significantly higher accuracy in variable retrieval (92.62%) compared to direct prompting of the LLM on the full patient chart without structural constraints (54.02%). Sensitivity averaged 81.49%, and specificity was high at 95.34%, excelling in ruling out noncomplications. Cohen's κ of 0.86 indicates substantial agreement and robust performance for complex tasks.

### Performance of Human Manual Chart Review

Manual chart review required an average of 25.2 minutes per chart, totaling 38.2 hours over 15 days, with an overall accuracy of 82.28% (Fig. 2). While human reviewers performed well on basic structured tasks, such as identifying surgical dates (88.67% accuracy), they struggled with complex or unstructured data, achieving only 78.92% accuracy for advanced tasks like detecting postsurgical complications. In comparison, OphthoACR demonstrated superior accuracy, achieving 96.48% for basic tasks and 92.62% for advanced tasks.

### Performance of AI-Assisted Chart Review

AI-assisted chart review significantly improved both accuracy and efficiency in data extraction compared to manual review. The overall accuracy of AI-assisted review was 97.07%, with task-specific performance reaching 98.90% for basic tasks, 96.71% for intermediate tasks, and 95.60% for advanced tasks (Fig. 2), reducing the error rate by 15% compared to manual review. Notably, OphthoACR was able to correct expert ophthalmologists by accurately identifying overlooked postoperative complication variables in 77.78% of cases where its findings differed from initial human review. The two most common circumstances in which AI successfully identified errors missed by clinicians were (1) when patients had multiple visits following secondary IOL surgery, increasing the “noise” when identifying a complication or ocular value, and (2) when data included subtle indicators of postoperative complications that were more likely to be overlooked, such as “trace” anterior chamber inflammation.

The review process was also faster in AI-assisted review, averaging 8.8 minutes per chart (13.5 total hours), a 64.9% reduction from the 25.2 minutes per chart (38.2 total hours) required for manual review (Fig. 3). To note, the time taken for OphthoACR does not take into account time taken for pre- and postprocessing time, which is not needed in manual human review. We acknowledge that although these steps will only slightly increase OphthoACR runtime, they are not needed steps in manual review. Overall, integration of AI assistance into manual review led to significant improvements in both the accuracy of data retrieval and the speed of the review process, providing a reliable and efficient augmentation to manual chart review.

## Discussion

OphthoACR represents a groundbreaking tool in fully automating the end-to-end process of retrospective chart review, transforming unstructured patient charts into highly accurate, detailed individual patient chart reviews and cleaned cohort-wide data sets, without the need for manual intervention. Achieving an overall accuracy of 94.25%, including 92.62% for higher-complexity tasks like evaluating postoperative complications, OphthoACR sets a new benchmark for automated clinical data interpretation.

OphthoACR significantly reduces chart review time, processing each chart in an average of 80 seconds compared to 25.5 minutes for manual review, while also generating detailed timelines for ocular values and complications that manual reviews lack (Supplementary Fig. S2). Its ability to process multiple charts in parallel allows entire cohorts to be reviewed simultaneously, transforming a once weeks-long task into one completed in minutes. This efficiency is one of the standout features of OphthoACR's pipeline, as parallel processing is critical for scaling of large cohort analyses.

Uniquely versatile, OphthoACR is capable of extracting longitudinal patient outcomes and visualizing postoperative trends in ocular parameters and complications. By consolidating key temporal findings into clear visual summaries, our tool provides clinicians with a comprehensive view of patient histories to support informed decision-making. Furthermore, OphthoACR's ability to cite specific chart locations for its outputs fosters trust by enabling real-time fact-checking by physicians. In addition to analyzing modern EHR data, OphthoACR also processes legacy records, including handwritten notes, offering a complete and accurate review of patient histories, including those before EMR.

### OphthoACR Is Scalable and Adaptable

OphthoACR offers critical advantages over current in-house NLP tools, particularly in scalability, efficiency, and generalizability.^10^ In-house models, such as those presented by Senders et al. and Kaur et al., rely on institution-specific data for training, limiting their ability to perform effectively across different health care settings.^6^^,^^30^ Despite substantial investments, these models often struggle to generalize, showing reduced accuracy when applied to new clinical environments.^14^

In contrast, OphthoACR leverages OpenAI's GPT-4, which has been pretrained on a vast and diverse data set of publicly available and licensed data, as its baseline model, enabling it to handle complex medical terminology and accurately interpret unstructured text in clinical notes.^15^ Applying RAG and domain-specific fine-tuning, we enhance GPT-4’s versatility to create a model tailored for conducting automated chart reviews in ophthalmology.^16^ By adjusting RAG parameters and fine-tuning inputs, OphthoACR can be seamlessly adapted to other institutions and even different medical specialties with minimal effort.^13^^,^^17^ Additionally, GPT-4’s commercial availability promotes collaboration and reproducibility, overcoming the limitations of proprietary in-house NLPs that may quickly become obsolete as new technology arises while driving adoption across diverse settings.^18^^,^^19^

### Human Component of AI-Assisted Review Versus Fully Automated AI Review

Fully automated chart reviews excel in large-scale data analysis, offering efficiency for identifying trends, flagging discrepancies, and monitoring outcomes across patient populations. This approach is ideal when time and resources are limited, enabling effective quality control without the demands of manual review.

AI-assisted chart reviews combine the scalability of automation with the nuanced expertise of clinicians, making them invaluable for cases that require human validation. OphthoACR uniquely provides precise references for its conclusions in chart locations. Clinicians can quickly validate findings, such as flagged complications or trends, with clear contextual information, including text snippets and temporal labeling. As one expert ophthalmologist noted, “With OphthoACR, I can see not only what was flagged but also where it was found and why, making validation much faster and easier.” This hybrid approach empowers clinicians to focus on critical decision-making while minimizing time spent on data retrieval.

### Study Limitations

This study has limitations. Using intervalidated manual chart reviews as the ground truth may introduce bias due to human error. OphthoACR currently cannot handle cases with multiple surgeries in a short time frame, risking misattributed complications, and may lack the nuanced insight required for certain clinical scenarios, best assessed by physicians. The study was limited to 91 patients from a single institute, with future research needed on larger, more diverse cohorts to assess portability. Potential implementation challenges include computational costs, technical expertise requirements, and privacy concerns, emphasizing the need for robust PHI-compliant infrastructure and governance for secure, ethical use.^20^^,^^21^ In terms of model costs, for OphthoACR, at the time this study was conducted, running approximately 2000 words of a patient's chart across our 20 variables currently costs about $0.20 per chunk. We are continuing to explore cost-efficient strategies, including alternative LLMs and GPT API batch processing, while acknowledging that model costs remain dynamic as the technology rapidly evolves.^22^ Model drift and performance variability are also important to note. LLMs like OphthoACR can change over time as newer versions of its base OpenAI are released, likely leading to improved reproducibility and reliability. As such, we have documented the version and model date of the LLM we used but acknowledge that access to the LLM may become defunct over time. Finally, as it stands, LLMs like GPT-4 and OphthoACR are purely tools, not approved by the US Food and Drug Administration, and should only be used with human-in-the-loop systems for verification.

### Ethical Implications

The utilization of OphthoACR and other automated AI-driven tools for EHR chart review introduces technical advantages and complex ethical challenges related to data privacy, security vulnerabilities, and adversarial attacks.^23^ Emerging research shows machine learning models are susceptible to cybersecurity threats, even after data sets are deidentified following HIPAA's Safe Harbor Law.^24^ A critical concern is the possibility of external or internal breaches of AI systems processing clinical data, which includes (1) exploitation of API endpoints, which allows unauthorized access to model outputs; (2) adversarial attacks by trained endpoint stealing models on RAG LLM models, as seen in Zheng et al.^25^; and (3) data poisoning by maliciously manipulating inputs to distort model behavior or bias outputs to create harmful or misleading interpretations.

Another critical ethical challenge of AI chart review is the potential for algorithmic bias. It is known that AI models trained on EHR data may perpetuate disparities in diagnostic accuracy or risk prediction, particularly among racial and ethnic minorities, who receive different patterns of care or have less complete documentation.^26^ This bias disproportionately affects certain patient populations. To mitigate these risks, it is recommended that API ownership groups like OpenAI conduct routine bias audits. These audits stratify model performance across demographic subgroups, correct for disparities in predictive accuracy, and reduce bias propagation.^27^ Finally, transparency is essential in this rapidly growing intersection of medical-technological innovation. Disclosing the use of AI tools in research and publications maintains trust and informs future oversight of algorithmic research in medicine.

To address model safety, particularly the risk of hallucinations—a well-documented concern in LLMs—we implemented multiple safeguards within our pipeline. Most notably, we employed a RAG framework that grounds the model's outputs in retrieved source text from the patient chart, minimizing reliance on the model's pretrained knowledge. In addition, we configured the model with a low temperature setting to reduce output variability and limit speculative generations. Throughout our evaluation, we did not observe true hallucinations; errors that did occur were attributable to contextual misinterpretations, such as extracting a date associated with a scheduled but unperformed procedure. To facilitate human oversight and error tracing, the pipeline outputs not only the extracted variable but also the corresponding source text and its location within the chart. This transparent linkage enables real-time verification and clinician validation. Although a fine-tuned LLM is central to our pipeline, its role is restricted to extracting structured data from retrieved context, further reducing hallucination risk. While our current fine-tuning is tailored to the documentation style of our institution, the approach is adaptable to other institutional EHR systems. We also note that, given the grounding effect of retrieval, the relative contribution of fine-tuning to model performance in this setting may be modest, an area we plan to investigate in future work.

### Big Data Applications and Future Directions of OphthoACR

OphthoACR's scalability unlocks opportunities for big data applications, enabling health care institutions to efficiently analyze vast data sets in an era of exponential data growth. Capable of processing thousands of patient records simultaneously, OphthoACR empowers researchers and clinicians to conduct large-scale studies on patient outcomes, complication rates, and treatment efficacy with unprecedented speed and accuracy. This capability is especially valuable for population health management, where analyzing temporal trends across extensive data sets informs data-driven clinical decisions.

The next phase of development will test OphthoACR on a large patient cohort of cataract surgery cases at CUIMC, encompassing both digitized and raw paper charts. This will validate its performance in real-world, high-volume clinical settings, demonstrating its ability to handle diverse patient histories and complex outcomes. Additionally, to further enhance evidence-based ophthalmic conclusions, we seek to further integrate medical and ophthalmologic knowledge bases into OphthoACR, including American academy of ophthalmology guidelines and EyeWiki.^28^^,^^29^

OphthoACR represents a transformative advancement in clinical data review in ophthalmology, tailoring state-of-the-art language models with advanced pre- and postprocessing systems to deliver fully automated end-to-end chart review. By reducing the labor-intensive process of chart review from weeks to minutes, OphthoACR establishes a new standard for ophthalmologic chart review and health care data utilization, which in the future may enable clinicians to manage higher patient volumes, conduct large-scale studies with unparalleled efficiency, and drive meaningful advancements in patient outcomes.

## Supplementary Material

Supplement 1

Supplement 2

Supplement 3
