# Supplementary figures and images for: OphthoACR (Ophthalmology Automated Chart Review): An AI-Powered Tool for Complete Automation of Ophthalmology Chart Reviews and Cohort Data Analysis
Source: Transl Vis Sci Technol. 2025 Oct 9;14(10):8. doi: 10.1167/tvst.14.10.8 (PMC12517364; doi:10.1167/tvst.14.10.8)

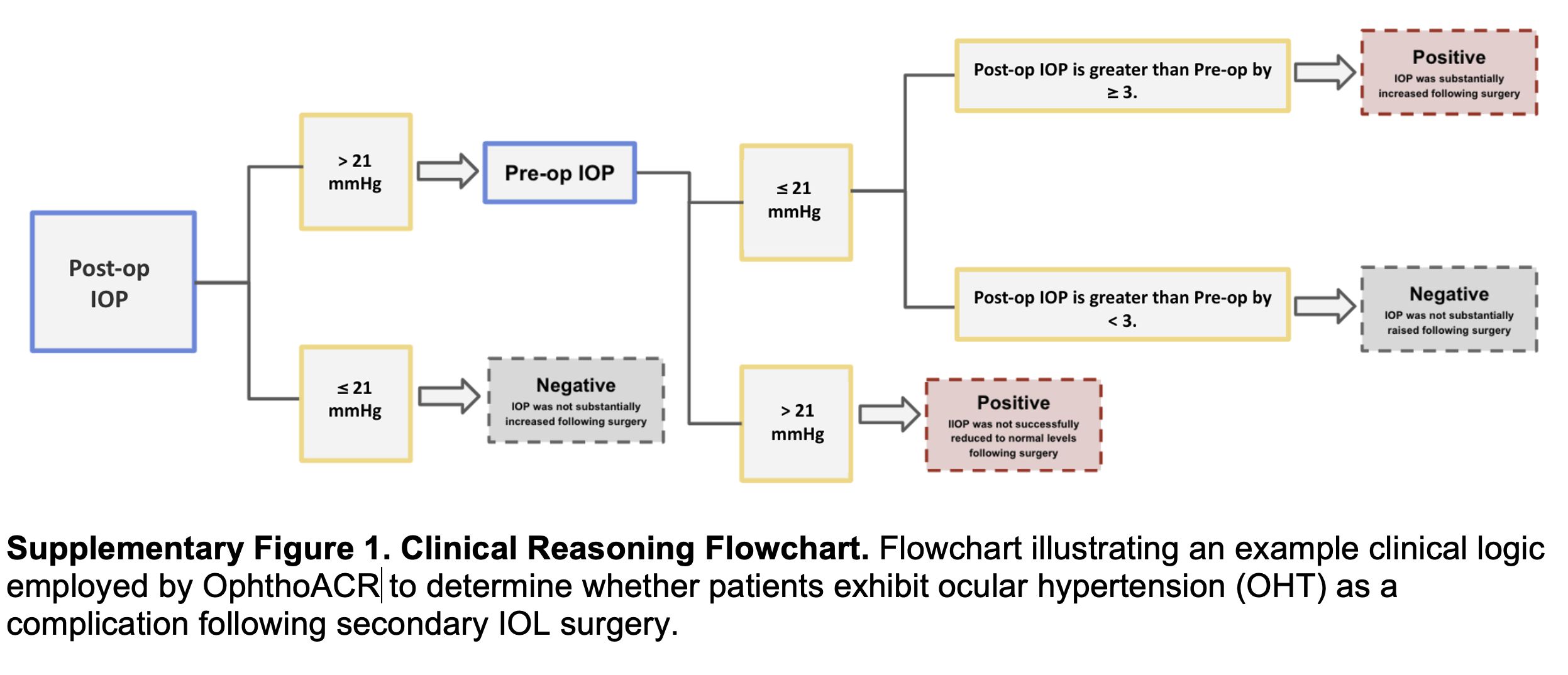

Supplement: Supplement 1 [file tvst-14-10-8_s001.jpg]

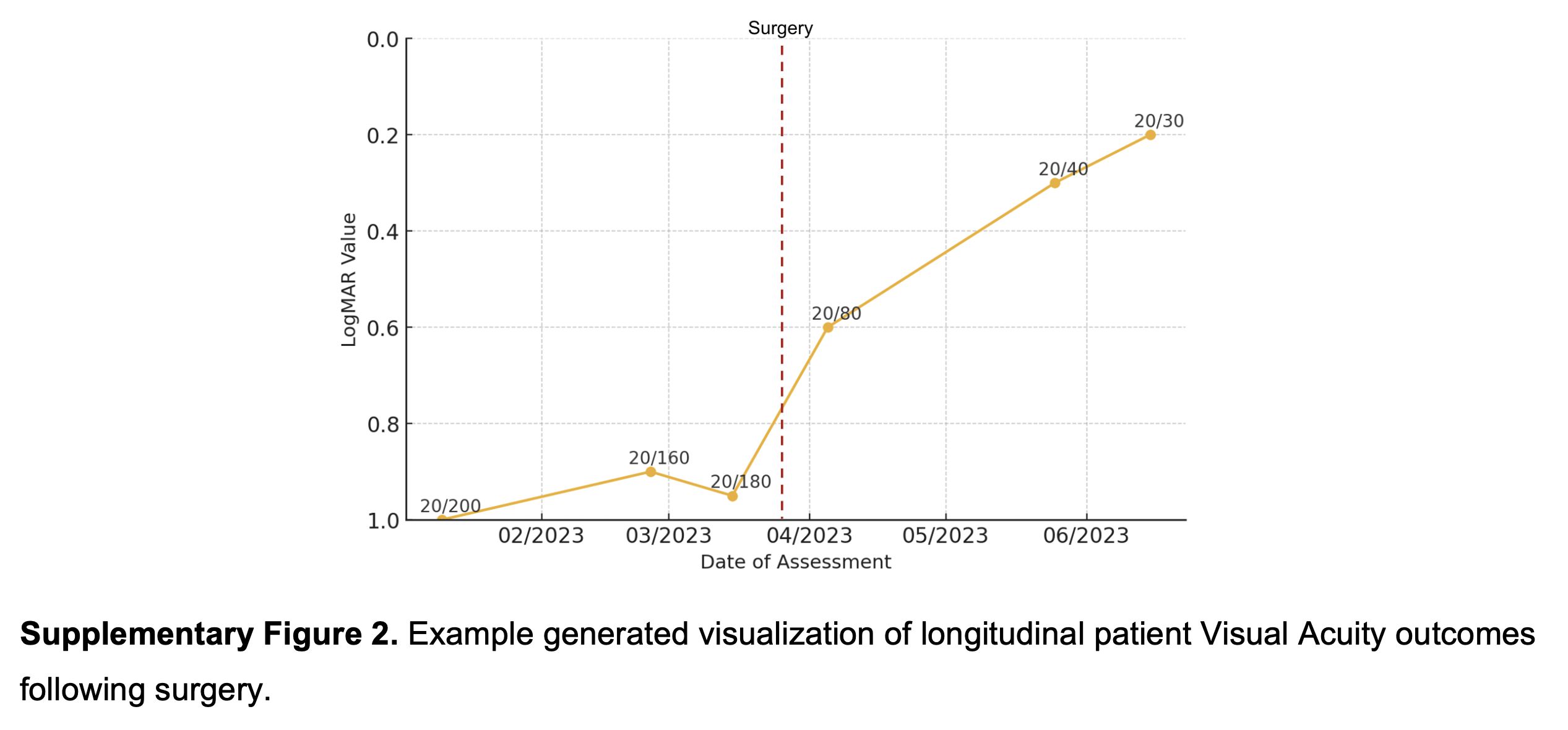

Supplement: Supplement 2 [file tvst-14-10-8_s002.jpg]
