# Supplementary material for: OphthoACR (Ophthalmology Automated Chart Review): An AI-Powered Tool for Complete Automation of Ophthalmology Chart Reviews and Cohort Data Analysis
Source: Transl Vis Sci Technol. 2025 Oct 9;14(10):8. doi: 10.1167/tvst.14.10.8 (PMC12517364; doi:10.1167/tvst.14.10.8)
Supplement: Supplement 3 [file tvst-14-10-8_s003.pdf]

Variable Definitions:

Simple:

- Operated eye:
  - Description: Indicates which eye received the secondary intraocular lens (IOL).
  - Example: "Right" or "Left."
- Date of surgery
  - Description: The date on which the secondary IOL surgery was performed.
  - Example: "2024-01-15."
- Date of initial cataract surgery
  - Description: The date when the initial cataract surgery took place.
  - Example: "2023-05-10."
- Model of lens
  - Description: The specific model or type of the secondary intraocular lens implanted.
  - Example: "CT Lucia 602" or "Akreos AO60"
- Implanted power
  - Description: The diopter power of the secondary IOL that was implanted.
  - Example: "21.5 D."
- Technique used
  - Description: The surgical technique used to implant the secondary IOL.
  - Example: "Scleral," "Sulcus," "Anterior Chamber," "Iris"

Intermediate:

- Visual acuity
  - Description: A temporal list of all measures of the patient's vision acuity.
  - Example: 20/25
- Axial length
  - Description: A temporal list of all measures of the patient's axial length.
  - Example: 24.5 mm.
- Keratometry
  - Description: A temporal list of all measures of the patient's axial length.
  - Example: 42.75 D / 44.00 D (K1 / K2)
- Refractive errors
  - Description: A temporal list of all measures of the patient's refractive error values (cyl, sph, axis).
  - Example: (0.5, -1.0, 152)

<u>Advanced</u>:

- Macular edema
    - Description: A temporal list of each time swelling or fluid accumulation in the macula was noted. Includes where in chart text the finding was found.
    - Example: "Present" or "Absent."
- Corneal edema
    - Description: A temporal list of each time swelling or fluid accumulation in the cornea was noted. Includes where in chart text the finding was found.
    - Example: "Present" or "Absent."
- Anterior chamber inflammation
    - Description: A temporal list of each time inflammation in the anterior chamber of the eye was noted. Includes where in chart text the finding was found.
    - Example: "0" or "4."
- Intraocular pressure
    - Description: A temporal list of all measures of the patient's IOP.
    - Example: 16 mmHg

<u>Etc:</u>

- Other comorbid diseases
    - Description: A temporal list of any underlying diseases the patient may have that can affect the eyes or surgery outcomes.
    - Example: "Diabetes mellitus" or "Marfan's Syndrome."
- Other eye surgeries
    - Description: A temporal list of all additional eye surgeries the patient has undergone, aside from the secondary IOL surgery.
    - Example: "Vitrectomy," or "Corneal transplant."

Data Standardization Procedure

Following OCR conversion and raw text extraction from the EHR, all clinical documents were subjected to systematic preprocessing using a custom Python-based pipeline. This step was critical to reducing heterogeneity in formatting, terminology, and structure across ophthalmology patient records. While not strictly required for the functioning of OphthoACR, this step plays an important role in improving model performance by reducing input noise and variability, ultimately enabling more accurate and consistent outputs. The entire standardization process consisted of the following components:

1. Whitespace and Line Break Normalization

Scanned and OCR-converted documents frequently contained irregular spacing, fragmented lines, excessive line breaks, and nonsensical filler characters introduced during type conversion, all of which hindered text interpretation. These artifacts were corrected using regular expressions to collapse unnecessary whitespace and newlines, ensuring that related phrases remained contiguous and interpretable by the model.

Example:
Original:
OD: 20/40 \n\n\n\n\n\n\n\n\n\n\

214&901248@$#

OS: 20/60

→

Standardized: *OD: 20/40 OS: 20/60*

2. Terminology and Abbreviation Normalization

Ophthalmology records often used inconsistent shorthand, abbreviations, and variable terminology. To address this, we developed a normalization dictionary of commonly encountered abbreviations (see below) and their standardized equivalents, applied using case-insensitive pattern matching. This ensured consistency across patients and providers. (OD, OS, VA were left as is)

- **OU** – Both Eyes
- **HM** – Hand Motion
- **LP** – Light Perception
- **NLP** – No Light Perception
- **CF** – Counting Fingers
- **Tn** – Tension (Intraocular Pressure)
- **IOP** – Intraocular Pressure
- **CME** – Cystoid Macular EdemaCys
- **RPE** – Retinal Pigment Epithelium
- **C/D** – Cup-to-Disc Ratio
- **CE** – Corneal Edema
- **ME** – Macular Edema
- **ACI** – Anterior Chamber Inflammation
- **OHT** – Ocular Hypertension

- **PSC** – Posterior Subcapsular Cataract
- **PVD** – Posterior Vitreous Detachment
- **RD** – Retinal Detachment
- **POAG** – Primary Open-Angle Glaucoma
- **IOL** – Intraocular Lens
- **AL** – Axial Length
- **Kmax** – Maximum Keratometry

To illustrate how this dictionary was applied, representative examples of raw, unstructured input text and their standardized counterparts are shown below:

Messy Input	Standardized Output
Tn 15/16	Intraocular Pressure: OD 15 mmHg, OS 16 mmHg
Hm os	OS Hand Motion
Ou ce sp	Both eyes Corneal Edema post-surgery
CF 3 Lt os	OS Counting Fingers at 3 feet
va r:20/40 l:20/50	Visual Acuity: OD 20/40, OS 20/50
pt HM - R	OD Hand Motion

3. Standardization of Tabular and Temporal Data

Longitudinal values such as visual acuity and intraocular pressure were frequently embedded in narrative text or ad hoc tables, often formatted inconsistently. These were extracted and rewritten into a line-item structure using ISO-8601 date format and consistent measurement labeling.

Example:

Original:

8/1: R 20/30 L 20/40; 9-15-23 OD:20/25 OS 20/30

→

Standardized:

2023-08-01: OD 20/30, OS 20/40
2023-09-15: OD 20/25, OS 20/30

This standardization protocol ensured that input data was converted into a coherent and consistent format suitable for high-throughput, semantically grounded analysis. While not required for the operation of OphthoACR, standardizing inputs is good practice, as it enhances clarity, structure, and reliability, ultimately improving the quality, accuracy, and interpretability of downstream outputs.

Confidence Heuristics for Best-of-n Sampling:

To identify the most reliable output among multiple completions generated via best-of-n sampling (n=5), we applied a confidence-based reranking framework using a predefined set of heuristics tailored to the structured clinical extraction task. Each candidate output was scored based on the following criteria: (1) Field completeness, evaluating whether all expected variables—such as intraocular pressure, visual acuity, and IOL power—were present and correctly formatted; (2) Consensus heuristics, if an output's value for a given variable deviated from the values reported in the majority of other completions, it was penalized, (3) Format conformity, verifying adherence to the defined JSON schema and absence of hallucinated or malformed content. In instances where multiple outputs received identical heuristic scores, the tie was resolved by selecting the output with the highest semantic overlap with the retrieved source text, thereby prioritizing groundedness and traceability to the patient chart.

Prompt Schema:

The provided snippet represents an altered example of a structured prompt schema used to guide the lLLM in extracting visual acuity data from ophthalmology clinical text. This schema defines the target function (extract_visual_acuity) and its associated parameters, each with a type constraint and description. Retrieved content from the RAG pipeline is embedded into the prompt, enabling contextual grounding. This schema ensures that model outputs are consistent, interpretable, and machine-readable.

```
□...

messages = [
```

```
    {
        "role": "system",
        "content": (
            "You are an expert clinical reviewer specializing in ophthalmology. "
            "You will extract structured data from patient chart text, following the exact output schema provided."
        )
    },
    {
        "role": "user",
        "content": (
            "Here is the relevant chart text retrieved from the patient's record:\n\n"
            "CHART EXCERPT:\n"
            "12/01/2023: VA (sc): OD 20/40, OS 20/100. IOP: OD 14, OS 16. \n"
            "Patient reports improved vision since last visit.\n\n"
            "Extract the visual acuity values using the following format."
        )
    },
    {
        "role": "function",
        "name": "extract_visual_acuity",
        "parameters": {
            "date": {"type": "datetime", "description": "Date of measurement."},
            "value": {"type": "string", "description": "Visual acuity value."},
            "eye": {"type": "string", "description": "OD or OS."},
            "evidence": {"type": "string", "description": "Exact chart text supporting this value."}
        }
    }
```

]

...

□This schema is passed to the LLM during structured prompting and allows for precise and constrained data extraction aligned with clinical variables of interest.

Post Processing:

JSON Cleaning and Standardization

All extracted JSON data underwent a rigorous post-processing cleaning process by script to:

- Remove invalid values and inconsistencies in data formatting.
    - Invalid values included physiologically impossible measurements (e.g., intraocular pressure < 3 mmHg or > 80 mmHg, visual acuity formats not conforming to Snellen or logMAR standards), malformed dates, or missing units. These were flagged using conditional thresholds and regular expressions, then either re-run or manually flagged for review.
- Format units correctly for seamless comparisons (e.g., standardizing intraocular pressure values).
    - "IOP: 18/20" → clarified and reformatted as "IOP: OD 18 mmHg, OS 20 mmHg"
- Convert key ophthalmic variables into standardized formats to ensure consistency across preoperative and postoperative analyses:
    - Visual acuity (VA): Converted to logMAR.
    - Keratometry readings (K1, K2): Standardized as Kmax.
    - Intraocular lens (IOL) power: Spherical, cylindrical, and axis values formatted uniformly.
        - Formatting was standardized using rule-based regular expressions that identified common variations (e.g., "+21.5D SPH", "CYL -1.25", "Axis: 090"), which were then converted to a unified schema: {(Sph: 21.50, Cyl: -1.25, Axis: 90.)}.
    - Anterior chamber inflammation (ACI) gradings: Standardized for easier categorization.

Post-Operative Ocular Parameter Outcomes and Complication Detection

For temporal variables, a script was used to ensure accurate extraction of these values. The script retrieved the clinically relevant postoperative value closest to desired timepoint (e.g. 3 months post-surgery) from the output list of all temporally labeled variables.

Additionally, we developed custom complication detection algorithms that simulate clinician reasoning by flagging abnormalities within predefined postoperative timeframes (e.g., 3–6 months post-op). For example:

- Ocular Hypertension (OHT): An IOP reading > 21 mmHg at 3 months post-op was flagged.
- Corneal Edema (CE): Identified based on documented mentions of corneal thickening, Descemet's folds, or epithelial changes. Complication present if CE persists after 3 months.

This adaptive logic ensures that the extracted insights closely align with clinical decision-making processes. An example of a logical workflow using OHT can be seen in Supplementary Figure 1.

Cohort-Wide Analysis

Once individual chart review extractions were complete, OphthoACR automatically conducted cohort-wide analyses of key ophthalmic variables, such as cohort-wide improvement in visual acuity. This final step ensured that standardized, structured outputs were available for immediate clinical review, offering timely insights into overall surgical outcomes and complication trends across the study population.

Error Handling

Lastly, the pipeline incorporated robust error-handling mechanisms, which included re-running the query or flagging for manual review, to address issues such as:

- Malformed JSON outputs (e.g., syntax errors, missing fields).
- Inconsistencies in structured fields (e.g., invalid visual acuity).
- Failed or incomplete extractions, triggering retry mechanisms to prevent workflow interruptions or failures.
